# Machine Learning Approach for Preterm Birth Prediction Using Health Records: Systematic Review

**DOI:** 10.2196/33875

**Published:** 2022-04-20

**Authors:** Zahra Sharifi-Heris, Juho Laitala, Antti Airola, Amir M Rahmani, Miriam Bender

**Affiliations:** 1 Sue & Bill Gross School of Nursing University of California Irvine, CA United States; 2 Department of Computing University of Turku Turku Finland

**Keywords:** preterm birth, prediction model, machine learning approach, artificial intelligence

## Abstract

**Background:**

Preterm birth (PTB), a common pregnancy complication, is responsible for 35% of the 3.1 million pregnancy-related deaths each year and significantly affects around 15 million children annually worldwide. Conventional approaches to predict PTB lack reliable predictive power, leaving >50% of cases undetected. Recently, machine learning (ML) models have shown potential as an appropriate complementary approach for PTB prediction using health records (HRs).

**Objective:**

This study aimed to systematically review the literature concerned with PTB prediction using HR data and the ML approach.

**Methods:**

This systematic review was conducted in accordance with the PRISMA (Preferred Reporting Items for Systematic Reviews and Meta-Analyses) statement. A comprehensive search was performed in 7 bibliographic databases until May 15, 2021. The quality of the studies was assessed, and descriptive information, including descriptive characteristics of the data, ML modeling processes, and model performance, was extracted and reported.

**Results:**

A total of 732 papers were screened through title and abstract. Of these 732 studies, 23 (3.1%) were screened by full text, resulting in 13 (1.8%) papers that met the inclusion criteria. The sample size varied from a minimum value of 274 to a maximum of 1,400,000. The time length for which data were extracted varied from 1 to 11 years, and the oldest and newest data were related to 1988 and 2018, respectively. Population, data set, and ML models’ characteristics were assessed, and the performance of the model was often reported based on metrics such as accuracy, sensitivity, specificity, and area under the receiver operating characteristic curve.

**Conclusions:**

Various ML models used for different HR data indicated potential for PTB prediction. However, evaluation metrics, software and package used, data size and type, selected features, and importantly data management method often remain unjustified, threatening the reliability, performance, and internal or external validity of the model. To understand the usefulness of ML in covering the existing gap, future studies are also suggested to compare it with a conventional method on the same data set.

## Introduction

### Background

Preterm birth (PTB), a common pregnancy complication, is responsible for 1.085 million (35%) of the 3.1 million neonatal deaths each year and significantly affects approximately 15 million children annually worldwide [1]. Survivors often suffer from lifetime disabilities, including motor function problems, learning disabilities, and visual and hearing dysfunctions [2]. In almost all high- and middle-income countries, PTB and its adverse consequences are the major leading causes of death in children aged <5 years [2]. According to the World Health Organization, PTB is defined as birth before 37 completed weeks of gestation (<259 days) from the first day of a woman’s last menstrual period. In general, there is a negative association between gestational age and poor pregnancy outcomes and long-term complications such as hospitalization, longer stay in the neonatal intensive care unit, and death [2]. Long-term hospitalization and frequent medical services required for PTB survivors may lead to additional mental distress and extra costs for the family, and it also imposes more strain on the health care system [3]. Current screening tests for PTB prediction can be categorized into three main groups: (1) risk factor evaluation, (2) cervical measurement, and (3) biochemical biomarker assessment. However, not all approaches have potential to be translated into clinical predictive utility, safely and cost-effectively [4]. They may also be insufficient for detecting true-positive PTB cases. For example, biochemical assessment is a costly procedure that may impose physical and mental stress to the pregnant individual. Risk factor assessment is another commonly used approach for which information comes from evidence-based practice that is an end outcome of statistical hypothesis testing (often including 1 factor to be tested) under controlled settings, which is a time- and money wasting approach. The latter may also leave behind many potential risk factors that did not receive researchers’ attention, advancing to hypothesis testing. By contrast, previous PTB history is one of the dominant risk factors, with a relative risk of 13.56, leaving nulliparous women undetected [3,5]. These findings indicate the insufficiency of the current methods in predicting high-risk pregnancies, specifically in those who are experiencing their first pregnancy. A few predictive systems have also been studied using series of information including maternal demographics, medical and obstetrical history, and well-known risk factors; unfortunately, however, their predictive power has been very limited [6,7]. This limitation may be because they often rely on simple linear statistical models that lack the capacity to model complex problems such as PTB. It is suggested that risk factor assessment using conventional approaches is insufficient, as >50% of PTB pregnancies will fail to be identified [8]. Thus, identifying additional screening tools for covering the gap in conventional prediction approaches is highly critical, as it helps guide prenatal care and prepare for potential early interventions required for poor prognosis. Recently, machine learning (ML) methods have been applied to further improve individual risk prediction beyond traditional models. Many ML methods can model the complex nonlinear relationships between the predictor features and the outcome. ML techniques can learn the structure from data without being explicitly programmed for its function [9]. For the ML approach, a significant volume of data is required to create robust models with high accuracy.

### Objectives

Fortunately, health records (HRs) in most countries contain data regarding one’s sociodemographic, obstetric, and medical history. This makes HRs appropriate data sets for ML models to learn and eventually predict the intended outcome. There has been growing research on applied ML on HR data to identify efficient predictive models for the early diagnosis of PTB. Few systematic or literature reviews, although are informative, are not focused on PTB [10]. This systematic review article aims to review the literature that has attempted to use ML on HR data to predict mothers who are at risk for PTB.

## Methods

### Overview

This systematic review was conducted in accordance with the PRISMA (Preferred Reporting Items for Systematic Reviews and Meta-Analyses) statement. A comprehensive search was performed in bibliographic databases including PubMed, CINAHL, MEDLINE, Web of Science, Scopus, Engineering Village (Compendex and Inspec), and IEEE Computer Society Digital Library, until May 15, 2021, in collaboration with a medical librarian (Stephen L Clancy). The search terms included controlled and free-text terms. The search strategy and number of articles found from each database are shown in Multimedia Appendix 1. Two review authors (ZSH and JL) independently performed the title or abstract and full-text screening. Potential disagreements were resolved by a third independent researcher. Nonrelevant articles were excluded in the title and abstract screening, and for the full-text article screen, reasons for exclusion per article were recorded. References of the identified articles were also checked for potential additional papers. Data were extracted by ZSH and confirmed by JL. Discrepancies were revisited by both authors to guarantee the database accuracy.

### Eligibility Criteria and Study Selection

Studies were included if they aimed to predict PTB risk by using HR data. The outcome variable was PTB occurrence, which is globally defined as any pregnancy termination between 20 and 37 weeks of gestation. Although in some studies PTB was defined differently in terms of age range, all definitions were aligned under 37 weeks of gestational age. The PTB definition serves to examine and establish model performance (ie, the ability of the intended model to distinguish PTB cases from non-PTB cases). The papers were required to include a statement of the ML domain or any of its synonyms. To identify any study that failed to include a ML statement in the title or abstract, an extensive list of commonly used ML model techniques was added to the search strategy.

### Selection Process

Selected articles were peer reviewed in the Covidence web-based software [11] by 2 independent reviewers. To assess relevancy, all studies were screened based on titles, abstracts, and full texts in two steps. In the first step, the abstracts of all articles gathered from the databases were screened in terms of their relevance to our study aim. Next, those articles with relevant titles or abstracts resulting from the first step underwent a full-text assessment. To resolve the raised disagreement, a third reviewer was involved for consulting. All articles that were concerned with heart rate variability assessment during pregnancy were included.

### Quality of Evidence

The quality of studies was assessed using the criteria proposed by Qiao [12]. Although the criteria proposed by Qiao [12] were too restrictive, no other quality assessment tool was found for the quality assessment of the studies. In this approach, quality assessment is based on five different categories: unmet needs, reproducibility, robustness, generalizability, and clinical significance. Unmet needs are met if the limits were reported in current non-ML approaches (eg, current methods have low diagnostic accuracy). A study is considered reproducible if it describes used feature engineering methods, platforms and packages, and hyperparameters. The condition for robustness is fulfilled if valid methods are used to overcome the overfitting (*k*-fold cross-validation or bootstrap*:* when a data set is large, splitting it into separate training, validation, and test sets is the best approach [13], and *k*-fold cross-validation and bootstrap are required only with the small data sets when there are not enough data for a 3-way split [14]) and the stability of results (variation of the validation statistic) are reported. The generalizability condition is met if the model is validated using external data. A study is considered to have clinical significance if predictors are explained and clinical applications for the model are suggested. Quality assessment was conducted by providing a *yes* or *no* response for each of the 5 categories. However, in our study, we attempted to be more descriptive; thus, a short description was provided for some of the criteria when applicable in the quality assessment table (Table 1).

**Table 1:** Quality assessment.

Study	Unmet need (existing gap)	Reproducibility				Robustness			Generalizability (external validation data)		Clinical significance	
		Feature engineering	Platform package	Hyperparameters	Valid methods to overcome overfitting		Stability of results			Predictor explanation		Suggested clinical use
Weber et al, 2018 [15]	Yes	Yes	Yes	No	5-fold CV^a^		Minimum and maximum values reported from the CV	No		Logistic regression coefficients and odds ratios		No
Rawashdeh et al, 2020 [16]	Yes	Yes	Yes	Number of neighbors for KNN^b^, number of hidden layers for ANN^c^, number of trees for RF^d^	Train-test split. Train size 237 with 19 positives. Test size 37 with 7 positives		No	No		No		Yes
Gao et al, 2019 [17]	Yes	Representing medical concepts as a bag of words and word embeddings, TF-IDF^e^, discretization of continuous features	No	No	Train-test split. Train size 17,607 with 132 positives. Test size 8082 with 85 positives		Minimum and maximum values and CIs	No		Feature importance, odds ratio		Yes
Lee and Ahn, 2019 [18]	Yes	No	Yes	Only neural network architecture described	Train-test split. Both train and test sets contained 298 participants		No	No		Feature importance (RF and ANN)		No
Woolery and Grzymala-Busse, 1994 [19]	Yes	No	Yes	No	A total of 3 different data sets used in isolation; 50-50 train-test split was used with each data set		No	No		No		No
Grzymala-Busse and Woolery, 1994 [20]	Yes	No	Yes	No	A total of 3 different data sets used in isolation; 50-50 train-test split was used with each data set		No	No		No		No
Vovsha et al, 2014 [21]	Yes	No	Yes	No	Data separated timewise to 3 data sets, and 80-20 train-test split was used with each data set; 5-fold CV to select models		No	No		Feature importance (linear SVM^f^)		No
Esty et al, 2018 [22]	Yes	No	Yes	No	No		No	No		No		No
Frize et al, 2011 [23]	Yes	No	Yes	No	Division into 3 data sets (parous and nulliparous). Train-test-verification splits		SDs of the metrics were reported	No		No		No
Goodwin and Maher, 2000 [24]	Yes	No	Yes	No	Train-test split (75%-25%)		No	No		Feature importance		No
Tran et al, 2016 [3]	Yes	Unigrams were created from free-text fields after removal of stop words	No	No	Train-test split (66%-33%)		No	No		Feature importance		Yes
Koivu and Sairanen, 2020 [9]	Yes	New features were created. Continuous features were standardized, and nominal features were one-hot encoded	Yes	All hyperparameters described	Data set partitioned into 4 parts (feature selection, training, validation, and test, with stratified splits of 10%-70%-10%-10%)		95% CIs for metrics	Yes		Feature importance		Yes
Khatibi et al, 2019 [25]	Yes	Imputation with mode for categorical features and median for continuous features	No	No	Train-test split		No	No		Feature importance		No

^a^CV: cross-validation.

^b^KNN: *K*-nearest neighbor.

^c^ANN: artificial neural network.

^d^RF: random forest.

^e^TF-IDF: term frequency-inverse document frequency.

^f^SVM: support vector machine.

### Data Synthesis

The reviewed studies were not homogenous in terms of methodology and data set; thus, a meta-analysis was not possible. A narrative synthesis was chosen to bring together broad knowledge from various approaches. This type of synthesis is not the same as a narrative description that accompanies many reviews. To synthesize the literature, we applied a guideline from Popay et al [26]. The steps included (1) preliminary analysis, (2) exploration of relationships, and (3) assessment of the robustness of the synthesis. Theory development was not performed because of the exploratory nature of the research synthesized. Thematic analysis was applied to extract the main themes from all the studies. The two main themes developed in the results represent the main areas of knowledge available regarding ML models applied for PTB prediction during pregnancy. These included *descriptive characteristics of the data set* (eg, data source, population, case and control definition, and feature selection) and *ML methodologies* (eg, feature selection, model processing, performance evaluation, and findings). We could not compare the studies because of the divergence of studies in terms of data set, ML model processing, and evaluation metric. The quality of the papers was assessed using the method proposed by Qiao [12].

## Results

### Study Selection

**Figure 1:**
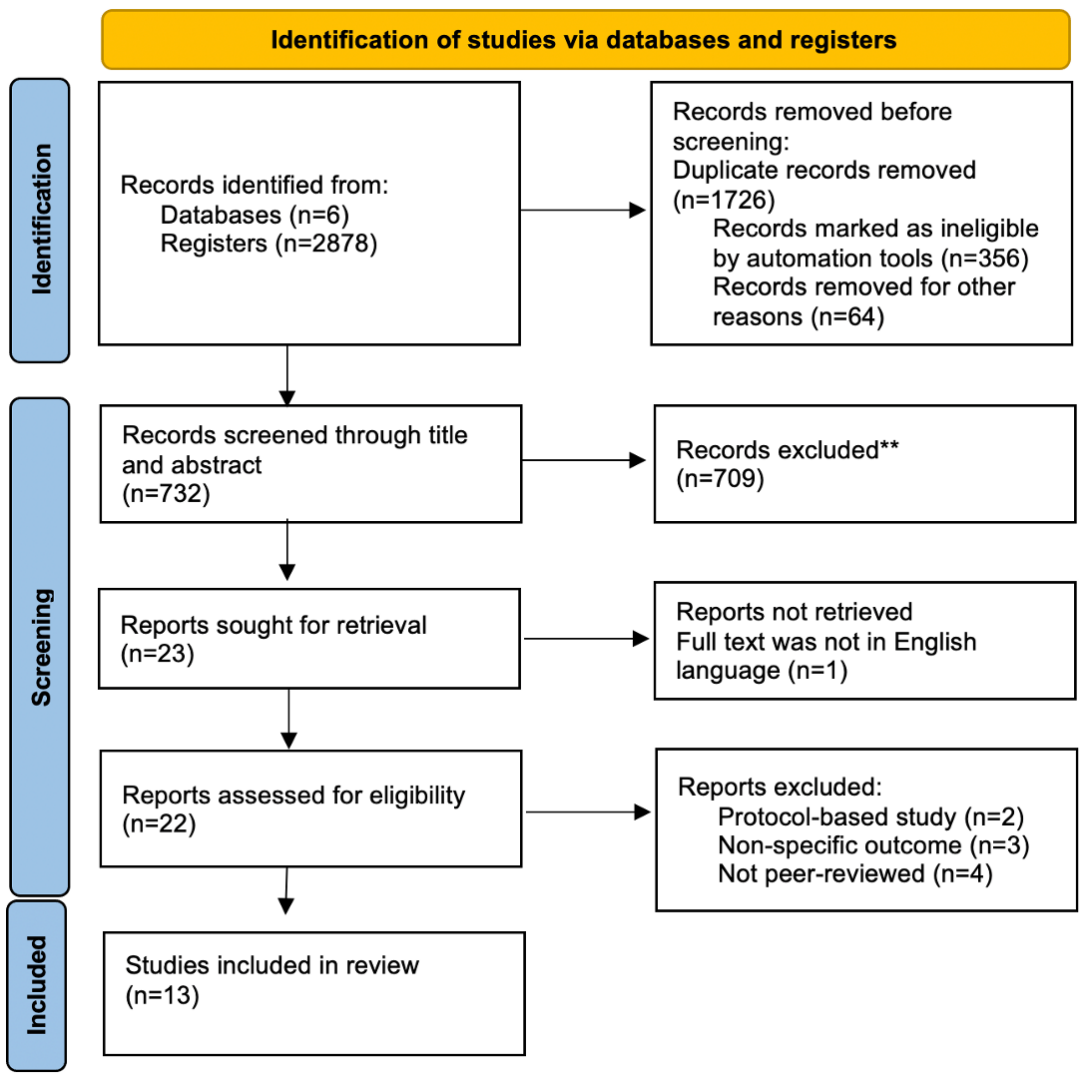
PRISMA (Preferred Reporting Items for Systematic Reviews and Meta-Analyses) chart.

After removing duplicates, 732 papers were screened through title and abstract. Of these 732 studies, 23 (3.1%) were screened by full text, resulting in 13 (1.8%) papers that met the inclusion criteria. Reasons for exclusion at this stage were recorded and are shown in the flow diagram in Figure 1.

### Study Characteristics

All the studies were retrospective and used one or more data sets recorded in clinical settings. Of the 13 studies, 7 (54%) were conducted in or after 2018 and 9 (69%) originated from the United States. The time length for which data were extracted varied from 1 to 11 years, and the oldest and newest data were related to 1988 and 2018, respectively. Of the 13 studies, 6 (46%) did not report the ethnicity or race of the population whose data were modeled. Various data sets were used for the studies, and the number of data sets varied from 1 to 3 in each study. The types of information included in each data set varied, including demographic, obstetric history, medical background, and clinical and laboratory information. Demographic information was included in almost all of the data sets used in the included studies. The size of the population whose data have been used for ML modeling varied from 274 to 13,150,017 people, and the number of features considered for modeling varied from 19 to 5000 depending on the data set used. PTB was defined differently from study to study; the cutoff point for the control and study groups (PTB and non-PTB) was defined as the 37th week of gestational age for 77% (10/13) of the studies that matched the standard cutoff point between term and PTBs. Of the 13 studies, 3 (23%) determined the PTB cutoff based on the frequency of the newborn death [17], newborn viability chance [16], or no justification [20]. It was not always specified whether abortion (pregnancy termination <20 weeks) was included in the models. Indeed, there was often no clear discernment of abortion and PTB in the reviewed studies (see Table 2 for more details).

**Table 2:** Descriptive characteristics of studies and feature selection.

Study, country, and type of study	Population characteristics	Data source (number of features)	Population (birth)	Study (PTB^a^), control groups, and type of PTB	Feature selection process and gestational week for when selected features are related	Number of selected features	Date
Weber et al, 2018 [15], United States, retrospective	Nulliparous women with a singleton birth (<32, ≥20, and ≥37 weeks); non-Hispanic Black (n=54,084) and White (n=282,130)	Birth certificate and hospital discharge records: >1000 features	336,214	PTB (early spontaneous): ≥20 and <32 weeks; control: ≥37 weeks	Factors with uncertain and ambiguous values were excluded, highly correlated features were collapsed, exclusion of features with no variation; —^b^	20	2007 to 2011
Rawashdeh et al, 2020 [16], Australia, retrospective	Australian; pregnancies with cervical cerclage	Data from a fetal medicine unit in a tertiary hospital in NSW^c^: 19 features	274	PTB (spontaneous): <26 weeks; control: >26 weeks	Unnecessary features (eg, medical record numbers) were excluded	19	2003 to 2014
Gao et al, 2019 [17], United States, retrospective	Caucasian (>68%), Black (16%-21%), and other (10%-13%)	EHR^d^ of Vanderbilt University Medical Center: 150 features	25,689	PTB: <28 weeks; control: ≥28 weeks; type of PTB was not distinguished	Features were arranged by their information gain and top 150 features were retained; —	150	2005 to 2017
Lee and Ahn, 2019 [18], Korea, retrospective	Korean; induced labors were excluded	Anam Hospital in Seoul	596	PTB (spontaneous): >20 and <37 weeks; control: ≥37 weeks	—	14	2014 to 2018
Woolery and Grzymala-Busse, 1994 [19], United States, retrospective	—	3 data sets: 214 features in total	18,890	PTB: <37 weeks; control: ≥37 weeks; type of PTB was not distinguished	—	Data set 1 (n=52), data set 2 (n=77), and data set 3 (n=85)	1994
Grzymala-Busse and Woolery, 1994 [20], United States, retrospective	—	3 data sets:153 features in total	9480	PTB: <36 weeks; control: ≥36 weeks; type of PTB was not distinguished	—	Data set 1 (n=13), data set 2 (n=73), and data set 3 (n=67)	1994
Vovsha et al, 2014 [21], United States, retrospective	—	NICHD^e^-MFMU^f^ data set: >400 features	2929	PTB (spontaneous and induced): <32, <35, and <37 weeks; control: ≥37 weeks	Logistic regression with forward selection, stepwise selection, LASSO^g^, and elastic net; —	24th week (n=50), 26th week (n=205), and 28th week (n=316)	1992 to 1994
Esty et al, 2018 [22], United States and Canada, retrospective	—	BORN^h^ and PRAMS^i^: 520 features	782,000	PTB: <37 weeks; control: ≥37 weeks; type of PTB was not distinguished	Features with >50% missing values were removed before missing value imputation; features come from before the 23rd gestational week	520	—
Frize et al, 2011 [23], United States, retrospective	—	PRAMS: >300 features	>113, 000	PTB: <37 weeks; control: ≥37 weeks; type of PTB was not distinguished	Decision tree (to establish consistency between data sets, features specific to the United States were excluded, eg, Medicaid and Women Infants Children Program); features come from before the 23rd gestational week	19 for parous and 16 for nulliparous	2002 to 2004
Goodwin and Maher, 2000 [24], United States, retrospective	—	Duke University’s Medical Center TMR TM perinatal data: 4000−5000 features	63,167	PTB: <37 weeks; control: ≥37 weeks; type of PTB was not distinguished	Heuristic techniques (features related to week <37 were included); —	32 demographic and 393 clinical	1988 to 1997
Tran et al, 2016 [3], Australia, retrospective	Australian	RNS^j^, NSW	15,814 births	PTB (spontaneous and elective): <34 and <37 weeks; control: ≥37 weeks	Features kept based on their importance (top *k* features; [27]); the rare features that occur in <1% of data points were removed; features come from before the 25th gestational week	10	2011 to 2015
Koivu and Sairanen, 2020 [9], United States, retrospective	White, Black, American Indian or Alaskan native, and Asian or Pacific Island individuals	CDC^k^ and NYC^l^ data sets	13,150,017	PTB: <37 weeks; control: ≥37 weeks; type of PTB was not distinguished	Excluding highly correlated features with correlation analysis (Pearson); —	26	CDC: 2013 to 2016; NYC: 2014 to 2016
Khatibi et al 2019 [25], Iran, retrospective	Iranian	National maternal and neonatal records (IMaN^m^ registry): 112 features	>1,400,000	PTB (spontaneous and medically indicated): >28 and <37 weeks; control: ≥37 weeks	Parallel feature selection and classification methods including MR_-_PB-PFS (features with nonzero scores are selected as top features); —	112	2016 to 2017

^a^PTB: preterm birth.

^b^Not reported in the study.

^c^NSW: New South Wales.

^d^EHR: electronic health record.

^e^NICHD: National Institute of Child Health and Human Development.

^f^MFMU: Maternal-Fetal Medicine Units Network.

^g^LASSO: least absolute shrinkage and selection operator.

^h^BORN: Better Outcomes Registry Network.

^i^PRAMS: Pregnancy Risk Monitoring Assessment System.

^j^RNS: Royal North Shore.

^k^CDC: Centers for Disease Control and Prevention.

^l^NYC: New York City.

^m^IMaN: Iranian Maternal and Neonatal Network.

### Data Selection

Of the 13 studies, 9 (69%) reported at least one piece of preprocessing information regarding the included data. The preprocessing step included data mapping, missing data management, and the class imbalance management in data. For the feature selection, of the 13 studies, 11 (85%) reported at least one method for the feature selection process. The number of features selected for each study varied from 10 to 520 for final ML modeling. On the basis of the literature surveyed, of the 13 studies, only 2 (15%) used unsupervised feature selection. In addition, of the 13 studies, 3 (23%) did not use feature selection, and some studies did use some heuristics instead. Owing to the divergency in feature selection, we could not identify clear trends on how the used approach would affect the model performance (see Table 3 for more information).

**Table 3:** Data processing and machine learning modeling.

Study	Preprocessing data			Model		Dominant model		Evaluation metrics		Analysis software and package		Findings
	Missing data management	Class imbalance										
Weber et al, 2018 [15]	MICE^a^	—^b^	Super learning approach using logistic regression, random forest, *K-*nearest neighbors, LR^c^ (LASSO^d^, ridge, and an elastic net)		No difference between models		Sensitivity, specificity, PVP^e^, PVN^f^, and AUC^g^		Rstudio (version 3.3.2), SuperLearner package		AUC=0.67, sensitivity=0.61, specificity=0.64	
Rawashdeh et al, 2020 [16]	Instances with missing values were removed manually	SMOTE^h^	Locally weighted learning, Gaussian process, K-star classifier, linear regression, *K*-nearest neighbor, decision tree, random forest, neural network		Random forest		Accuracy, sensitivity, specificity, AUC, and G-means		WEKA^i^ (version 3.9)		Random forest: G-mean=0.96, sensitivity=1.00, specificity=0.94, accuracy=0.95, AUC=0.98 (oversampling ratio of 200%)	
Gao et al, 2019 [17]	—	Control group were undersampled	RNNs^j^, long short-term memory network, logistic regression, SVM^k^, Gradient boosting		RNN ensembled models on balanced data		Sensitivity, specificity, PVP, and AUC		—		AUC=0.827, sensitivity=0.965, specificity=0.698, PVP=0.033	
Lee and Ahn, 2019 [18]	—	—	ANN^l^, logistic regression, decision tree, naïve Bayes, random forest, SVM		No difference between models		Accuracy		Python (version 3.52)		No difference in accuracy between ANN (0.9115) with logistic regression and the random forest (0.9180 and 0.8918, respectively)	
Woolery and Grzymala-Busse, 1994 [19]	—	—	LERS^m^		—		Accuracy		ID3^n^, LERS CONCLUS		Database 1: accuracy=88.8% accurate for both low-risk and high-risk pregnancy. Database 2: accuracy=59.2% in high-risk pregnant women. Database 3: accuracy=53.4%	
Grzymala-Busse and Woolery,1994 [20]	—	—	LERS based on the *bucket brigade algorithm* of genetic algorithms and enhanced by partial matching		—		Accuracy		LERS		Accuracy=68% to 90%	
Vovsha et al, 2014 [21]	—	Oversampling techniques (Adasyn)	SVMs with linear and nonlinear kernels, LR (forward selection, stepwise selection, L1 LASSO regression, and elastic net regression)		—		Sensitivity, specificity, and G-means		Rstudio, glmnet package		SVM: sensitivity (0.404 to 0.594), specificity (0.621 to 0.84), G-mean (0.575 to 0.652); LR: sensitivity (0.502 to 0.591), specificity (0.587 to 0.731), G-mean (0.586 to 0.604)	
Esty et al, 2018 [22]	Imputation with the *missForest* package in R	Not clear	Hybrid C5.0 decision tree−ANN classifier		—		Sensitivity, specificity, and ROC^o^		R software, missForest Package, FANN^p^ library		Sensitivity: 84.1% to 93.4%, specificity: 70.6% to 76.9%, AUC: 78.5% to 89.4%	
Frize et al, 2011 [23]	Decision tree	—	Hybrid decision tree–ANN		—		Sensitivity, specificity, ROC for P^q^ and NP^r^ cases		See5, MATLAB Neural Ware tool		Training (P: sensitivity=66%, specificity=83%, AUC=0.81; NP: sensitivity=62.8%, specificity=71.7%, AUC=0.72), test (P: sensitivity=66.3%, specificity=83.9%, AUC=0.80; NP: sensitivity=65%, specificity=71.3%, AUC=0.73), and verification (P sensitivity=61.4%, specificity=83.3%, AUC=0.79; NP: sensitivity=65.5%, specificity=71.1%, AUC=0.73)	
Goodwin and Maher, 2000 [24]	PVRuleMinerl or FactMiner	—	Neural networks, LR, CART^s^, and software programs called PVRuleMiner and FactMiner		No difference between models		ROC		Custom data mining software (Clinical Miner and PVRuleMiner, FactMiner)		No significant difference between techniques. Neural network (AUC=0.68), stepwise LR (AUC=0.66), CART (AUC=0.65), FactMiner (demographic features only; AUC=0.725), FactMiner (demographic plus other indicator features; AUC=0.757)	
Tran et al, 2016 [3]	—	Undersampling of the majority class	SSLR^t^, RGB^u^		—		Sensitivity, specificity, NPV^v^, PVP, F-measure, and AUC		—		SSLR: sensitivity=0.698 to 0.734, specificity=0.643 to 0.732, F-measure=0.70 0.73, AUC=0.764 to 0.791, NPV=0.96 to 0.719, PVP=0.679, 0.731; RGB: sensitivity=0.621 to 0.720, specificity=0.74 to 0.841, F-measures=0.693 to 0.732, NPV=0.675 to 0.717, PVP=0.783 to 0.743, AUC=0.782 to 0.807	
Koivu and Sairanen, 2020 [9]	—	—	LR, ANN, LGBM^w^, deep neural network, SELU^x^ network, average ensemble, and weighted average WA^y^ ensemble		—		AUC		Rstudio (version 3.5.1) and Python (version 3.6.9)		AUC for classifiers: LR=0.62 to 0.64; deep neural network: 0.63 to 0.66; SELU network: 0.64 to 0.67; LGBM: 0.64 to 0.67; average ensemble: 0.63 to 0.67; WA ensemble: 0.63 to 0.67	
Khatibi et al, 2019 [25]	Map phase module	—	Decision trees, SVMs and random forests, ensemble classifiers		—		Accuracy and AUC		—		Accuracy=81% and AUC=68%	

^a^MICE: Multiple Imputation by Chained Equations.

^b^Not reported in the study.

^c^LR: linear regression.

^d^LASSO: least absolute shrinkage and selection operator.

^e^PVP: predictive value positive.

^f^PVN: predictive value negative.

^g^AUC: area under the ROC curve.

^h^SMOTE: Synthetic Minority Oversampling Technique.

^i^WEKA: Waikato Environment for Knowledge Analysis.

^j^RNN: recurrent neural network.

^k^SVM: support vector machine.

^l^ANN: artificial neural network.

^m^LERS: learning from examples of rough sets.

^n^ID3: iterative dichotomiser 3.

^o^ROC: receiver operating characteristic.

^p^FANN: Fast Artificial Neural Network.

^q^P: parous.

^r^NP: nulliparous.

^s^CART: classification and regression tree.

^t^SSLR: stabilized sparse logistic regression.

^u^RGB: Randomized Gradient Boosting.

^v^NPV: net present value.

^w^LGBM: Light Gradient Boosting Machine.

^x^SELU: scaled exponential linear unit.

^y^WA: weighted average.

### Identified Potential Risk Factors

Although the included features somewhat differed in the studies, some features were commonly used and considered potential risk factors that may predict PTB occurrence (Table 4).

**Table 4:** Frequency of potential risk factors in the studies (n=13).

Potential risk factors	Studies, n (%)
Previous PTB^a^	10 (77)
Hypertensive disorders	9 (70)
Maternal age	7 (54)
Cervical or uterus disorders (cerclage, myoma, or inconsistency)	7 (54)
Ethnicity and race	6 (46)
Diabetes (eg, gestational, mellitus)	6 (46)
Smoking or substance abuse	5 (38)
Multiple pregnancy	5 (38)
Education	4 (30)
Physical characteristics (BMI, weight, and height)	4 (30)
Parity	4 (30)
Marital status	3 (23)
Other chronic diseases (thyroid, asthma, systemic lupus erythematosus, or cardiovascular)	3 (23)
PTB symptoms (bleeding, contractions, premature rupture of membranes, etc)	3 (23)
Insurance	2 (15)
Income	2 (15)
In vitro fertilization	2 (15)
Stress or domestic violence	2 (15)
Infections (gonorrhea, syphilis, chlamydia, or hepatitis C)	1 (7)
Biopsy	1 (7)

^a^PTB: preterm birth.

### ML Modeling and Performance Assessment

Various basic and complex ML modeling approaches were used with different frequencies, including artificial neural network, logistic regression, decision tree, support vector machine (SVM) with linear and nonlinear kernels, linear regression (least absolute shrinkage and selection operator [LASSO], ridge, and elastic net), random forest, locally weighted learning, gradient boosting, learning from examples of rough sets, Gaussian process, K-star classifier, and naïve Bayes (Multimedia Appendix 2).

Although most studies reported the type of software applied for the ML analysis, only few of them specified the package they have used for the analysis. Several evaluation measures were used to assess the proposed models. These include sensitivity, specificity, area under the receiver operating characteristic curve, accuracy, predictive value positive, predictive value negative, G-mean, F-measure, and net present value, based on the frequency they have been used in the studies. Owing to the divergent methodology used for outcome assessment and model processing, comparison between models was not possible. However, overall, studies with a cutoff gestational age of 37th week, regardless of the model used, often showed lower sensitivity (40%-69%), except for 1 study that showed a sensitivity of 93% [22]. Those with an earlier cutoff gestational age of 26th to 28th weeks indicated higher sensitivity (96%-100%).

### Quality Assessment

In general, reviewed studies had satisfactory quality (Table 1). However, there was substantial variation, as some studies fulfilled almost every category, whereas others met only a few. All studies fulfilled *the unmet need category*, as PTB prediction is still an unsolved problem. Feature engineering was mentioned in almost half (6/13, 46%) of the studies [3,9,15-17,25]. Platforms and packages were not mentioned in 23% (3/13) of the studies [3,17,25]. Hyperparameters were described in only 23% (3/13) of the studies [9,16,18]. According to the criteria proposed by Qiao [12], of the 13 studies, only 1 (8%) used valid methods (*k*-fold cross-validation) to overcome overfitting [15]. However, many of the studies have population sizes of tens of thousands or higher, which makes the standard train-test split a valid approach for model evaluation, and there was no need for *k*-fold cross-validation. There is no commonly agreed criterion for sufficiency of data for a single train-test split to be sufficient, as this depends on factors such as number of features, relative sizes of the classes, and amount of noise in the data. As an example, previously, Kohavi [28] studied the accuracy estimation and model selection with the test set size of 500 instances as the lower limit for a single train-test split being considered reliable. In 23% (3/13) of the studies, the use of *k*-fold cross-validation or bootstrap instead of the train-test split would have been clearly the better choice because of the small population size (n<3000) [16,18,21]. The stability of the results is reported only for 31% (4/13) of the studies [9,15,17,23]. Of the 13 studies, only 1 (8%) used external validation data and met the requirement for generalizability [9]. Predictor explanation was provided in 62% (8/13) of the studies [3,9,15,17,18,21,24,25]. Only 31% (4/13) of the studies clearly suggested a clinical application for their method [3,9,16,17]*.*

## Discussion

### Principal Findings

Premature birth remains a public health concern worldwide. Survivors experience substantial lifetime morbidity and mortality rates. The conventional methods of PTB assessment that have been used by clinicians seem to be insufficient to identify PTB risk in more than half of the cases. The conventional methods that are concerned with health data (HR) are often statistical modeling, in which, first, input predictive factors are selected by a researcher and, second, the multifactorial nature of PTB is ignored. Thus, these methods suffer from biases and linearities. The linear vision on HR in conventional approaches is perhaps one of the major barriers to advancing our understanding of nonlinear interaction dynamics between potential risk factors of multifactorial PTB. ML modeling, in contrast to statistical modeling, investigates the structure of the target phenomenon without preassumption on data, and automatically and thoroughly explores possible nonlinear associations and higher-order interactions (more than 2-way) between potential the risk factors and the outcome [29]. ML modeling is expected to discover novel patterns, not necessarily novel predictive features, which provide an opportunity to gain insight into the underlying mechanisms of multifactorial outcomes (in this case PTB), where existing knowledge is still insufficient for developing a thorough predictive system [29]. Over the past 26 years, 13 studies have been published, creating ML-based prediction models using HR data, with the number of studies increasing over time.

Among the reviewed studies, the performance of various ML modeling indicated potential for predictive purposes. Owing to the different evaluation metrics used by studies, performance comparison across studies was not practical. On the basis of within-study synthesis, some studies compared nonlinear ML methods, such as deep neural networks, kernel SVMs, or random forests, to more basic linear models, such as logistic regression, LASSO, and elastic net. Of these 13 studies, 4 (31%) concluded that there was no significant difference between the predictive performances of the different applied methods [3,9,19,21]. For example, Tran et al [3] compared stabilized sparse logistic regression with randomized gradient boosting and found no significant differences between the methods. The conclusion that complex ML modeling is not superior to simple logistic modeling matches the findings of a recent systematic review conducted for a wider concept of clinical prediction. In the aforementioned review, Christodolou et al [30] compared the performance of logistic regression with more complex ML-based clinical prediction models; they found no evidence of the superior performance of the ML methods for clinical prediction. In contrast, some studies indicated a significant difference among various ML modeling approaches. For example, Rawashdeh et al [16] showed that random forest has a clear advantage over linear regression in predicting the week of delivery; however, the test set used in the study was very small for a reliable conclusion. Vovsha et al [21] also showed some improvements for nonlinear SVM over a linear model (linear SVM, LASSO, and elastic net) when classifying preterm versus full-term birth for the whole study population but did not find similar differences when making predictions for only spontaneous PTB or for first-time mothers. Gao et al [17] and Koivu and Sairanen [9] reported that deep learning–based approaches have better performance than logistic regression. The remaining studies did not include a comparison with a basic baseline method, such as logistic regression. In conclusion, these results imply that classical statistical models remain a competitive approach for predicting PTB. The current limitations of ML modeling and its infancy may explain its failure to cover the gaps in classical statistical models for PTB prediction using HR data. We suggest that more research is still required to ascertain with confidence whether ML methods, such as those based on deep learning, can systematically improve the predictive performance of the model as compared with basic statistical models.

An HR seems to be a useful data source, including the potential risk factors from which the ML model can learn the significant predictors as well as the nonlinear interaction among the identified risk factors.

A large sample size, as one of the distinct characteristics of HR data, is a double-edged sword that covers large populations but consumes time and requires advanced technology. A large data size can also be used to create validation sets. Most studies in this review had large sample sizes, including thousands of pregnant women. Although some studies performed internal validation, external validation was uncommon, and almost all studies validated the performance within the same HR. Th lack of external validity assessment limits generalizability and may reduce the discrimination validity of the model when applied in other sites and HR systems. External validation of the model through its application in a distinct data set may be helpful in understanding its usefulness and generalizability in different geographical areas, periods, and settings [31]. Furthermore, half of the studies in this review did not report the race or ethnicity of the population, which indicates ignoring the importance of the ethnic and health disparity in predictive model assessment. For example, ethnic minority groups, such as Black and Hispanic women, are more at risk of developing pregnancy complications, including PTB. Failure to consider ethnicity threatens the internal validity of ML modeling.

Large data sizes and reflective data types are as important as large sample sizes. HR data often appear insufficient to precisely identify risk factors that decrease the accuracy of predictive ML models. Indeed, small sample size and passive data that are limited to a few sociodemographic and medical histories seem insufficient to predict the multifactorial PTB. Enriched data that include more, time-sensitive, and dynamic characteristics of each individual (eg, life history, mental distress during various stages of pregnancy, and biomarker change) may increase the accuracy and integrity of the applied ML models. For example, being diagnosed with gestational diabetes is known to be a strong predictive factor for PTB among the features in ML models. However, owing to the dynamic nature of diabetes (glucose level), which can vary from moment to moment, particularly during pregnancy, applying a pool of data reflecting the dynamic glucose change in a person may be more accurate in predicting PTB in comparison with the presence or absence of diabetes. The difference in glucose change may also partially explain why some women with diabetes are at a higher risk of developing PTB. To achieve this accuracy in HR use, data should be enriched by more and dynamic features and ML models should be optimized to analyze the dynamic-natured potential risk factors that go beyond the clear-cut presence or absence of a feature [32].

In contrast, a small data size threatens the risk factor distinction for PTB prediction. There might be an indirect association between some predictive factors and PTB, falsifying the direct and actual associations. For example, smoking not only is introduced as a protective factor against mortality in low–birth weight and PTB infants but also is identified as a predictive factor for PTBs. In this case, PTB may not be the result of smoking directly itself but due to potential mediators, such as hypertension, which is triggered by smoking. Therefore, if there is no recorded information about blood pressure, the model may consider smoking as the actual risk factor. This highlights the importance of more possible health data to increase the ability of the ML model to distinguish between mediators and exposure features.

One of the major challenges in HR-based studies is the presence of missing data. Although missing data have been an acknowledged challenge in HR studies, a little more than half of the studies acknowledged the presence of missing data and a variety of analytic approaches to manage this absence. On average, despite its importance, there has been minimal work in this area, and it is unclear how such biased observations impact prediction models.

Another important challenge in HR-related models is unbalanced data between case and control groups. This problem is because PTB occurs in 10% of all births. Researchers have often applied oversampling techniques to handle unbalanced data. However, these techniques create artificial data that may not have much in common with actual observations. Oversampling techniques must be used carefully in validating models because if artificial instances end up in the test set (or test folds in cross-validation), one may obtain highly overoptimistic performance estimates.

In addition, all reviewed studies approached PTB prediction as a classification problem. There was often no clear discernment of abortion and PTB in the reviewed studies. This ambiguity, if it comes from missing to distinguish abortion from PTB in actual ML modeling, may threaten the specificity of the model in predicting PTB. In addition, as PTB and abortion have different leading causes, the findings of the studies may also be questionable. In addition, in the defined PTB time window (20-37 gestational week), classification remains problematic. In this case, neonates born at week ≤30 are considered to belong to the same class as those born at week 36 of pregnancy. However, the former is associated with a much higher risk of adverse outcomes and requires neonatal intensive care. Therefore, it could be more beneficial to approach PTB as a regression problem and try to predict the gestational age (as weeks or days) at childbirth. This approach could help identify PTB cases that have the greatest need for care.

### Conclusions

Overall, ML modeling has been indicated to be a potentially useful approach in predicting PTB, although future studies are suggested to minimize the aforementioned limitations to achieve more accurate models. Importantly, ML’s ability to cover the existing gap in conventional statistical methods remains questionable. To achieve reliable conclusions, our study suggests some considerations for future studies. First, more studies are needed to compare ML modeling with existing conventional methods in the same data set with the same amount of data and population. Conducting the comparison studies uncovers the potential superiority of one over the other. Second, the study population should be distinguished based on parity, particularly if previous pregnancy data were among the selected features. Otherwise, the model would probably rely on this strong predictive factor in multiparous women, leaving nulliparous women underserved and undetected. In addition, studies should be transparent to whether they use the same time frame for feature selection for case (PTB) and control (non-PTB) groups. For instance, assume that we have a cutoff point of 28 weeks before which we want our model to identify PTB cases. In this case, if we include the data for the control group to be after the cutoff point, which most likely differs from before the cutoff point, the model may rely on the information after the cutoff point for PTB prediction. Thus, the model fails to detect the cases before the specified time point. Third, two cutoff points should be clarified in model development: (1) the gestational cutoff week the study targets before the cases are detected and (2) the gestational time point before the features are selected. For example, Gao et al [17] determined the 28th week as the cutoff week before feature selection. However, it is not clear whether the created model would identify PTB before week 28, from where the features were collected, or any time before week 37, based on the data related to before the 28th week. The time interval between identified features and PTB occurrence, particularly if the PTB is symptomatic, can be more informative in terms of model specificity and time sensitivity in detecting symptomatic and asymptomatic PTB.

Enriched data size and optimized data type can also improve the usefulness of the ML model. Appropriate approaches for managing missing data and unbalanced control and case groups are also required to achieve more reliable and accurate results.

## Appendix

Multimedia Appendix 1 Search strategy.

Multimedia Appendix 2 Machine learning models’ frequency.

## Abbreviations

HR: health record
ML: machine learning
PRISMA: Preferred Reporting Items for Systematic Reviews and Meta-Analyses
PTB: preterm birth
SVM: support vector machine
