# Supplementary material for: Machine Learning Approach for Preterm Birth Prediction Using Health Records: Systematic Review
Source: JMIR Med Inform. 2022 Apr 20;10(4):e33875. doi: 10.2196/33875 (PMC9069277; doi:10.2196/33875)
Supplement: Multimedia Appendix 1 [file medinform_v10i4e33875_app1.docx]

Multimedia appendix1. Search strategy

| **Database** | **Search strategy** | **Filters** | **Articles#** |
| --- | --- | --- | --- |
| **PubMed 20210805** | ("Obstetric Labor, Premature"[Mesh] OR "Gestational Age"[Mesh] OR "preterm labor" OR "preterm labour" OR "premature labor" OR "premature labour" OR "preterm birth*" OR "premature birth*" OR "gestational age*") AND ("Artificial Intelligence"[Mesh] OR "artificial intelligence"[tiab] OR "computational intelligen*"[tiab] OR "computer intelligen*" OR "machine intelligen*"[tiab] OR "machine learn*"[tiab] OR "computer learn*"[tiab] OR "machine intelligen*"[tiab] OR "computer intelligen*"[tiab] OR "computer neural network*"[tiab] OR "computer reasoning"[tiab] OR "deep learning"[tiab] OR "hierarchical learning"[tiab] OR "supervised learning"[tiab] OR "support vector machine*"[tiab] OR "support vector network*"[tiab] OR "predictive model*"[tiab] OR "computer heuristic*" OR "expert system*"[tiab]) | English language | 594 |
| **Medline Complete 20210813 (corrected)** | (MH "Obstetric Labor, Premature") OR (MH "Gestational Age") OR ((preterm OR premature) N3 (labor OR labour OR birth*)) OR "gestational age*"  AND  (MH "Artificial Intelligence+") OR ((artificial OR computational OR computer* OR machine*)) N3 (intelligen* OR learn* OR "neural network*" OR reasoning OR heuristic*)) OR "deep learning" OR "hierarchical learning" OR "supervised learning" OR (("support vector") N3 (machine* OR network*)) OR "predictive model*" OR "expert system*" | English language | 740 |
| **CINAHL Complete 20210813 (corrected)** | (MH "Labor, Premature") OR (MH "Childbirth, Premature") OR (MH "Gestational Age") OR ((preterm OR premature) N3 (labor OR labour OR birth*)) OR "gestational age*  AND  (MH "Artificial Intelligence+") OR ((artificial OR computational OR computer* OR machine*)) N3 (intelligen* OR learn* OR "neural network*" OR reasoning OR heuristic*)) OR "deep learning" OR "hierarchical learning" OR "supervised learning" OR (("support vector") N3 (machine* OR network*)) OR "predictive model*" OR "expert system*" | English language | 245 |
| **Web of Science Core Collection 20210813** | ((preterm OR premature) NEAR/3 (labor OR labour OR birth*)) OR "gestational age*"  AND  ((artificial OR computational OR computer* OR machine*) NEAR/3 (intelligen* OR learn* OR "neural network*" OR reasoning OR heuristic*)) OR "deep learning" OR "hierarchical learning" OR "supervised learning" OR (("support vector") NEAR/3 (machine* OR network*)) OR "predictive model*" OR "expert system*"  **Databases searched within the Core Collection:** Science Citation Index Expanded (SCI-EXPANDED) --1900-present, Emerging Sources Citation Index (ESCI) --2005-present  **Databases excluded:** Social Sciences Citation Index (SSCI) --1900-present, Arts & Humanities Citation Index (A&HCI) --1975-present, Conference Proceedings Citation Index- Science (CPCI-S) --1990-present, Conference Proceedings Citation Index- Social Science & Humanities (CPCI-SSH) --1990-present, Book Citation Index– Science (BKCI-S) --2005-present, Book Citation Index– Social Sciences & Humanities (BKCI-SSH) --2005-present, Current Chemical Reactions (CCR-EXPANDED) --1985- present, Index Chemicus (IC) --1993-present.  (from Web of Science Core Collection)  You searched for: TOPIC: (((preterm OR premature) NEAR/3 (labor OR labour OR birth*)) OR "gestational age*") AND TOPIC: (((artificial OR computational OR computer* OR machine*) NEAR/3 (intelligen* OR learn* OR "neural network*" OR reasoning OR heuristic*)) OR "deep learning" OR "hierarchical learning" OR "supervised learning" OR (("support vector") NEAR/3 (machine* OR network*)) OR "predictive model*" OR "expert system*")  **Refined by:** LANGUAGES: ( ENGLISH ) AND [excluding] DOCUMENT TYPES: (PROCEEDINGS PAPER OR MEETING ABSTRACT OR EDITORIAL MATERIAL )  Timespan: All years. Indexes: SCI-EXPANDED, ESCI. |  | **455** |
| **Scopus 20210813** | ((preterm OR premature) W/3 (labor OR labour OR birth*)) OR "gestational age*"  AND  ((artificial OR computational OR computer* OR machine*) W/3 (intelligen* OR learn* OR "neural network*" OR reasoning OR heuristic*)) OR "deep learning" OR "hierarchical learning" OR "supervised learning" OR (("support vector") W/3 (machine* OR network*)) OR "predictive model*" OR "expert system*"  ( TITLE-ABS-KEY ( ( ( preterm OR premature ) W/3 ( labor OR labour OR birth* ) ) OR "gestational age*" ) AND TITLE-ABS-KEY ( ( ( artificial OR computational OR computer* OR machine* ) W/3 ( intelligen* OR learn* OR "neural network*" OR reasoning OR heuristic* ) ) OR "deep learning" OR "hierarchical learning" OR "supervised learning" OR ( ( "support vector" ) W/3 ( machine* OR network* ) ) OR "predictive model*" OR "expert system*" ) ) AND ( LIMIT-TO ( LANGUAGE , "English" ) ) AND ( EXCLUDE ( DOCTYPE , "cp" ) OR EXCLUDE ( DOCTYPE , "no" ) OR EXCLUDE ( DOCTYPE , "ed" ) OR EXCLUDE ( DOCTYPE , "cr" ) OR EXCLUDE ( DOCTYPE , "le" ) OR EXCLUDE ( DOCTYPE , "ch" ) OR EXCLUDE ( DOCTYPE , "sh" ) )  Doctypes: CP=Conference Paper, NO=Note, ED=Editorial,CR=Conference Review, LE=Letter, CH=Book Chapter, SH=Short Survey |  | 735 |
| **Engineering Village 20210813:** Includes Compendex (Engineering) and Inspec (physics, astronomy, electronics engineering, ocean engineering, geophysics, acoustics, computer science, mechanical and production engineering, biomedical controls, and information technology) | (artificial NEAR/3 intelligence) OR (computer NEAR/3 intelligence) OR (computers NEAR/3 intelligence) OR (computer NEAR/3 learning) OR (machine NEAR/3 learning) OR "deep learning" OR "hierarchical learning" OR "supervised learning" OR ("support vector" NEAR/3 machine) OR ("support vector" NEAR/3 network) OR "predictive model*" OR "expert system*"  AND  (preterm NEAR/3 labor) OR (premature NEAR/3 labor) OR (preterm NEAR/3 labour) OR (premature NEAR/3 labour) OR (preterm NEAR/3 birth) OR (premature NEAR/3 birth) OR "gestational age*" | English language | 72 Total (50 Compendex , 22 Inspec ) |
| **IEEE Computer Society Digital Library 20210813** | "preterm labor" OR "premature labor" OR "preterm labour" OR "premature labour" OR "preterm birth" OR "premature birth" OR "gestational age"  AND  artificial intelligence OR computer intelligence OR computer learning OR machine learning OR "deep learning" OR "hierarchical learning" OR "supervised learning" OR "support vector" OR "predictive model*" OR "expert system" OR "expert systems" | English language | 19 |
| **IEEE Xplore 20210816** | (((preterm OR premature) NEAR/3 (labor OR labour OR birth)) OR "gestational age") AND (((artificial OR computer OR machine) NEAR/3 (intelligence OR learning)) OR "deep learning" OR "hierarchical learning" OR "supervised learning" OR "support vector" OR "predictive model*" OR "expert system” |  | 18 |
