# Supplementary figures and images for: Machine Learning Approach for Preterm Birth Prediction Using Health Records: Systematic Review
Source: JMIR Med Inform. 2022 Apr 20;10(4):e33875. doi: 10.2196/33875 (PMC9069277; doi:10.2196/33875)

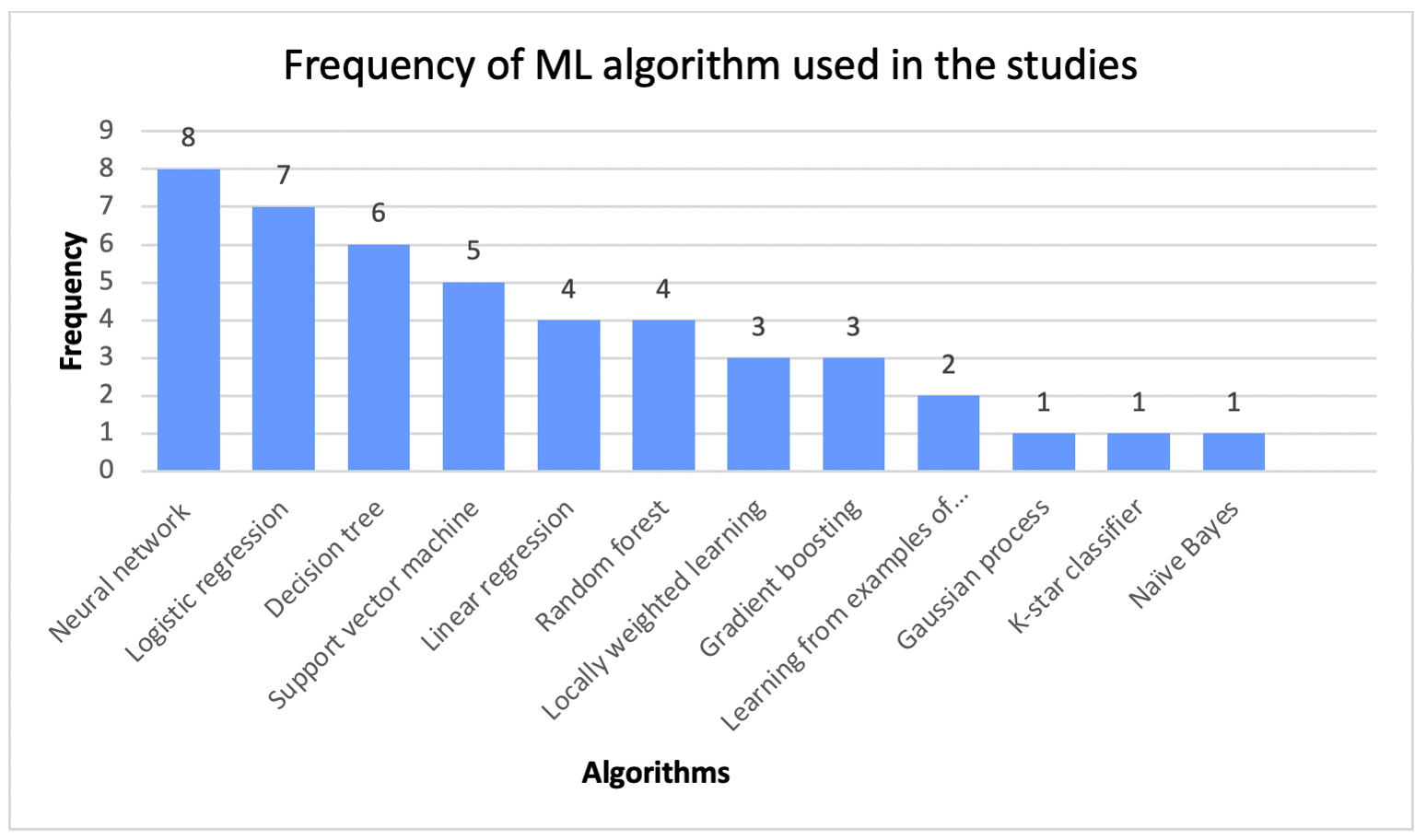

Supplement: Multimedia Appendix 2 [file medinform_v10i4e33875_app2.png]
